# Evidence for congruent impairment in micro and macrovascular function in type 1 diabetes

**DOI:** 10.1371/journal.pone.0187525

**Published:** 2017-11-13

**Authors:** Concetta Irace, Valentina Messiniti, Bruno Tassone, Claudio Cortese, Eugene J. Barrett, Agostino Gnasso

**Affiliations:** 1 Department of Health Science, Magna Græcia University of Catanzaro, Catanzaro, Italy; 2 Department of Experimental and Clinical Medicine, Magna Græcia University of Catanzaro, Catanzaro, Italy; 3 Department of Experimental Medicine and Surgery, Tor Vergata University, Rome, Italy; 4 Division of Endocrinology, Department of Medicine, University of Virginia, School of Medicine, Charlottesville, Virginia, United States of America; University of Illinois at Urbana-Champaign, UNITED STATES

## Abstract

Diabetes affects large and small vessels through mechanisms only partially known. In the present study, we evaluated the function of capillaries and large arteries in subjects with type 1 diabetes mellitus (T1DM) to study the effect of chronic hyperglycemia in the absence of other cardiovascular risk factors. Twenty-five subjects with T1DM and 12 healthy age-matched controls were enrolled. Nine patients had mild or moderate retinopathy. Contrast enhanced ultrasound was used to measure perfusion of the deep forearm flexor muscle of the non-dominant arm at rest (baseline) and after an ischemic stimulus (reactive hyperemia). Perfusion was expressed as Video Intensity (VI) in arbitrary unit (a.u.)/mm^2^. The time to reach peak VI after ischemia was also recorded. The function of large arteries was evaluated using flow-mediated vasodilation (FMD). VI was significantly lower in T1DM compared to control subjects both at baseline (0.22±0.16 vs 0.44±0.35 a.u./mm^2^, p<0.05), and after ischemia (0.33±0.24 vs 0.68±0.46 a.u./mm^2^, p<0.05). The time to reach peak VI after ischemia was markedly longer in T1DM (5.6±2.2 vs 4.0±1.7 seconds, p<0.02). These differences were more marked in T1DM subjects with retinopathy. FMD was lower in TIDM patients compared to controls (5.4±6.4 vs 10.7±4.5%, p<0.01). The present findings demonstrate that T1DM patients have defective peripheral skeletal muscle perfusion both at rest and after ischemia compared with control subjects. Low muscle perfusion associates with low FMD of the brachial artery. Furthermore, T1DM subjects with retinopathy have the least muscle perfusion and blunted response to hyperemia compared to T1DM without retinopathy.

## Introduction

Diabetes affects the function and morphology of blood vessels leading to increased cardiovascular morbidity and mortality[1]. Under physiological condition, blood vessels release vasoactive substances to regulate blood flow and meet tissue metabolic demand[2]. The vascular tree acts in a coordinated way: large arteries supply blood to small arteries and arterioles, which in turn adapt flow through the organs[3]. Capillaries, in spite of the small diameter, are the dominant site for the exchange of fluid and metabolites due to their large surface area, and have a key role in the homeostasis of glucose in the skeletal muscle. Indeed, muscle is responsible for about 80% of insulin-mediated glucose disposal. Insulin stimulates glucose uptake by increasing blood flow to the microvasculature, and glucose transport across the endothelium[4–7].

Diabetes adversely affects both large and small vessels. While non-invasive studies of large vessels in diabetic patients are abundant, non-invasive assessment of microvascular function (except in tissues like retina or skin) has been methodologically limited. Contrast Enhanced Ultrasound (CEU) is able to evaluate capillary perfusion in skeletal muscle *in-vivo* by assessing real-time blood volume within the microvasculature[8]. CEU makes use of contrast agents consisting of microbubbles of gases, encapsulated into a shell of different composition. The diameter of these microbubbles ranges between 2 and 8 micron, i.e. less than or equal to the diameter of a red blood cell. The difference in acoustic impedance between the gas in the microbubbles and the surrounding tissue *in-vivo* makes the microbubbles highly reflective resulting in the enhanced acoustic backscattering from blood[9].

CEU has been used to assess muscle microvascular function in healthy subjects and in subjects with metabolic syndrome, type 2 diabetes, and peripheral artery disease after provocative tests such as euglycemic hyperinsulinemic clamp or exercise[10–15]. Both type 2 diabetes and non-diabetic insulin-resistant states are associated with defective insulin-mediated muscle capillary recruitment as compared to healthy subjects. Insulin-resistance and circulating free fatty acids reduce insulin-mediated muscle capillary recruitment and glucose disposal, contributing to hyperglycemia[4]. Although relevant, *in-vivo* clinical studies of the microcirculation (other than in skin or retina) are scanty and more data are needed to evaluate the role of microvessels in the physiopathology of the vascular disease.

Most of the available data on *in-vivo* endothelial function are derived from the study of large arteries. Brachial artery reactivity measured by the flow-mediated dilation (FMD) technique has been extensively used to assess the ability of the endothelium to induce vasodilation after a transitory ischemic stimulus[16]. Defective brachial artery dilation is due to impaired synthesis and release of nitric oxide (NO), and is considered an early manifestation of atherosclerosis. It has been demonstrated that not only the reduction of vasodilation but also its delay is associated with cardiovascular risk factors (CVRFs)[17–20].

In the present research we evaluated the function of capillaries, using CEU, and of large arteries, using FMD, in otherwise healthy subjects with T1DM and age and sex comparable non diabetic healthy controls to study the effect of chronic hyperglycemia in the absence of other known CVRFs.

## Methods

### Study design and patients

This is a cross sectional study designed to evaluate the baseline perfusion and function of deep forearm flexor muscle vessels, and the function of the brachial artery using CEU and FMD technique in subjects with and without T1DM. Twenty-five consecutive subjects with T1DM who met the inclusion criteria and gave written informed consent to participate were enrolled. Inclusion criteria were: presence of Type 1 diabetes, HbA1c <9% (75 mmol/mol), and age>18 years. Exclusion criteria were: hypertension, hyperlipidemia, obesity, smoking habit, and previous cardiovascular events such as myocardial infarction, stroke, peripheral artery disease, and angina. We did not include these subjects in order to avoid the interference of vasoactive drugs on vascular function. All subjects with T1DM attending our outpatient clinic are screened on a regular basis for micro-(retinopathy, neuropathy, and kidney disease) and macro-vascular complications (cerebro-cardiovascular disease and peripheral arterial disease) according to the national current guidelines. Twelve healthy control subjects without diabetes or pre-diabetes, as documented by previous available blood testing, and comparable for sex, pre-menopausal state if females, and age (accepted difference ±5 years) were recruited among postgraduate students and hospital staff on a volunteer basis. Fasting blood glucose was measured at the enrollment and if ≥ 100 mg/dL subjects were excluded from the research. Additional exclusion criteria were family history of diabetes, and family history of cardiovascular disease. The research was approved by the Institutional Ethics Committee of the Policlinico Mater Domini of Catanzaro and conducted in accordance with the Declaration of Helsinki.

During the first visit body weight and height were recorded, the BMI calculated and blood sampled for HbA1c (only T1DM), fasting blood glucose and lipids (total cholesterol, HDL-cholesterol and triglycerides) measurements. Systolic (SBP) and diastolic blood pressure (DBP) were recorded twice on the right arm after 5 minutes of seated rest and the average used for the analyses. The medical history and current insulin treatment were recorded and a general physical exam performed. Subjects were invited for a second visit within 3 days, to perform the vascular study. They were also asked to fast overnight, and refrain from exercise and caffeine before the CEU and FMD test. T1DM subjects were further asked to refrain from breakfast insulin bolus, but to administer bed-time insulin the night before. Subjects on pump therapy maintained basal insulin infusion during the study. The FMD test was performed before CEU in order to avoid any interference on the Doppler signal by residual circulating microbubbles. All vascular studies were carried out in a temperature-controlled room at 22–24°C.

### Analytical methods

Blood glucose and lipids were measured with commercially available kits; HbA1c with high-performance liquid chromatography aligned with DCCT method.

### Real-Time contrast enhanced ultrasound study

**Fig 1:**
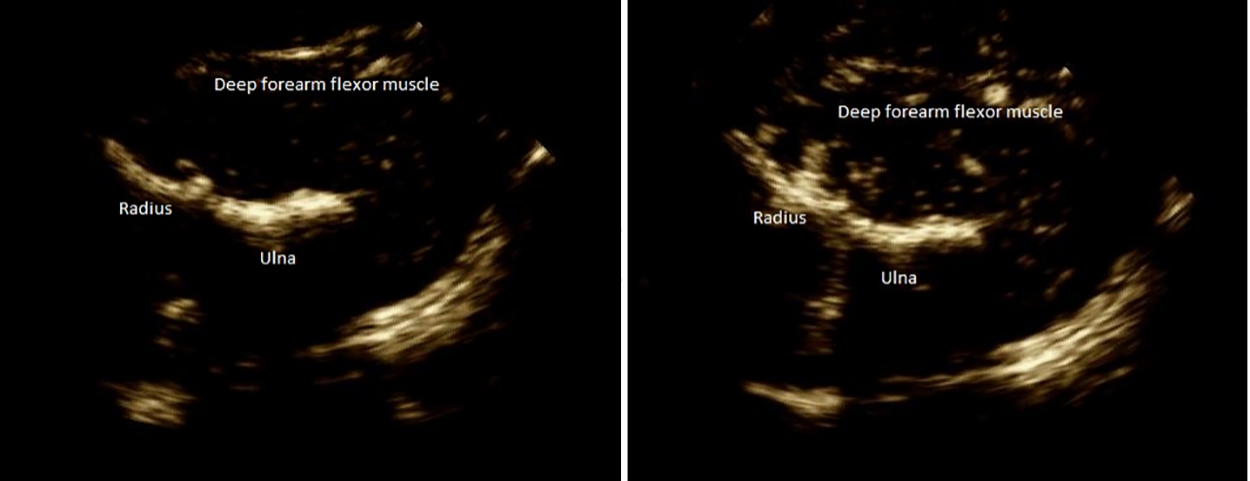
Deep forearm flexor muscle at baseline (Panel A) and after contrast media infusion (Panel B). Small white points detectable in Panel B are generated by reflection of microbubbles when insonated by ultrasound beam.

Real-Time CEU was used to measure muscle perfusion, expressed as Video Intensity (VI) (arbitrary unit, a.u./mm^2^), of the deep forearm flexor muscle of the non-dominant arm at baseline in resting condition, and after ischemic stimulus (reactive hyperemia) (Fig 1). Two-dimensional echography was performed using the echo-color-Doppler Philips HD 11 XE (Royal Philips Electronics, Netherlands) equipped with a 4–2 MHz sector phased array. A representative ultrasound slice of the flexor muscle in the upper third of the forearm, above and around the bone, was chosen. The transducer position was adjusted until a clear image of the muscle was obtained. The skin was marked, and the probe clamped in a probe holder in order to maintain position throughout the study. The phase inversion technology with low mechanical index (MI) of 0.18, and pulse repetition frequency (PRF) 2.5 Hz was used. Depth was set a 4–6 cm, one focus zone at 2–3 cm, dynamic range 232 dB, gain 60-75dB, and map B/2/2. A suspension of phospholipid-stabilized microbubbles filled with sulfur hexafluoride (SonoVue^®^, Bracco S.p.A., Milan, Italy) was infused at a rate of 1.2 ml/min into a large forearm vein through the antecubital fossa of the dominant arm. The mean diameter of these bubbles is 2.5 μm, and > 90% of the microbubbles are smaller than 8 μm[21]. The microbubble suspension was reconstituted from a 25 mg lyophilisate by addition of 5 ml of saline. The sulfur hexafluoride concentration was approximately 8 μl/ml. The suspension was then infused at a rate of 1.2 ml/min using a specific VueJect BR-INF 100 pump (Bracco S.p.A., Milan, Italy). The rate of infusion was determined in a preliminary study of 3 control subjects who underwent CEU four times using four different infusion rates (1, 1.2, 1.3, 1.5 ml/min), on four different days. The kit consisted of a 20Gx1” 1/4 cannula needle, 20 ml syringe, and a 910 mm length and 0.55 mm diameter catheter. The pump was let to oscillate regularly throughout the study in order to keep the microbubble suspension homogeneous. The protocol was designed to obtain the VI of the deep forearm flexor muscles in the baseline condition before ischemia (baseline-VI) after the steady state was obtained (~ 2 min), and the peak-VI after 1 min of ischemia recovery (peak-VI). To achieve forearm ischemia a pneumatic cuff, placed above deep forearm flexor muscle around the upper arm close to the elbow, was inflated up to 250 mmHg for 1 min.

Video clips with a frame rate “30/s” were acquired continuously during the study at baseline, during ischemia, and during reactive hyperemia (cuff release). The test lasted approximately 4 min. All the images were analyzed off line using the dedicated software QLab (Philips, SA, US). A large region of interest (ROI) of the imaged muscle was drawn avoiding bone, connective tissue and large vessels and copied into each file to ensure that the ROI was identical for each recording. We measured baseline-VI and peak-VI. The software automatically measured VI as arbitrary unit (a.u). We further adjusted VI for the ROI area (mm^2^) in order to compare VI between subjects with diabetes and healthy controls. We also measured the time to reach the peak-VI after cuff deflation. In addition to small vessels, we evaluated the perfusion of large vessels in the muscle using a preselected, automatically drawn, 1mm^2^ ROI. As for the small vessels, we evaluated baseline-VI, peak-VI, and the time to peak-VI. Percentage or Delta-VI increase during reactive hyperemia of small vessels of deep flexor muscles was calculated based on the following formula: Delta-VI (%) = {[(Peak-VI)–(baseline-VI) / (baseline-VI)] ×100}.

To evaluate the reproducibility of the method five volunteers were studied in four different occasions one week apart. The coefficient of variation (CV) was calculated as the ratio of the standard deviation to the mean. The CV was respectively 0.10 for the VI at baseline and 0.08 for the peak-VI.

### Flow Mediated Dilation (FMD) technique

The echo color Doppler Philips HD 11 XE (Royal Philips Electronics, Netherlands) equipped with a 12–3 MHz high-resolution linear array, steerable pulsed wave Doppler, and simultaneous ECG recording was used. FMD was evaluated in the brachial artery of the non-dominant arm. The artery was imaged ~10 cm above the elbow in the longitudinal section on the anterior side of the biceps muscle. The gain setting and the transducer position were adjusted until a clear image, showing near and far intima–lumen interface was obtained. The skin was marked, and the transducer clamped in a probe holder to keep the same position throughout the study. Internal diameter (ID) of the brachial artery was recorded at baseline and after the ischemic stimulus. ID was defined as the distance between intima–lumen interface of the near wall and lumen–intima interface of the far wall. Ischemia was induced by inflating a pneumatic cuff around the forearm up to 250 mmHg and maintaining inflation for 5 min. ID was measured off-line using dedicated software (Autodesk^®^ Design Review) before ischemia [baseline ID], and at 50 s, 2 min and 3 min after ischemia (reactive hyperemia), as previously reported[17]. FMD was expressed as percentage change from baseline and calculated using the following formula: {[(FMD-ID)–(baseline-ID)/ (baseline-VI)] ×100}. Based on the time of peak dilation, subjects were classified as ‘Early dilators’ if peak dilation occurred at 50s after ischemia; ‘Late dilators’ if peak dilation occurred after 50s and ‘No dilators’ if dilation did not occur after ischemia[17–20]. Peak FMD was defined as the maximal dilation measured at three different time points.

Brachial artery baseline systolic peak velocity (SPV), end diastolic velocity (EDV), and time average peak velocity (TAPV) were automatically recorded by the instrument as mean of the last three cardiac cycles. TAPV and baseline ID were used to calculate brachial artery blood flow (BF) using the following formula: BF = [cross-sectional Area (A)*TAPV]. Area was calculated as: A = [πx (ID^2^ x 0.785)] [22,23].

### Statistical analyses

Data were analyzed using SPSS 23 for Macintosh (SPSS, Inc., Chicago, IL). Based on available data, and the SD calculated in subjects enrolled to evaluate the reproducibility of the CEU method, for α = 0.05 and β = 0.10, the number of subjects to detect a clinically relevant difference of 5 a.u. VI was estimated to be 11 in each group.

Variables not normally distributed were BF, VI of large and small vessels (baseline and reactive hyperemia), and Delta reactive hyperemia-VI. The variables were normalized by the 2-Step Rank Transformation and parametric test applied. Triglycerides were also not normally distributed, therefore they were log-transformed before applying parametric test. The *t*-*test* for unpaired data and the chi square test were used to compare continuous variables and prevalence. The *t-test* for paired data was used to compare VI before and after ischemia in the same subjects. Differences among subjects divided according to the time of peak dilation (Early, Late and No dilators) were evaluated by ANOVA. The simple correlation was performed to evaluate the linear dependence between video intensity and continuous measured variables. The coefficient of determination R squared (R^2^) was calculated in order to evaluate the proportion of variance in video intensity for each variable. The multiple linear regression analysis was performed to evaluate which variables independently correlated with VI at baseline and after ischemia. Only variables significantly correlated to VI on simple regression analysis were included as independent variables (age, baseline brachial artery diameter, peak FMD), using the simultaneous method. Data have been expressed as unstandardized B coefficient and standardized β coefficient. The standard error for beta estimate has also been included in the relevant table.

## Results

Characteristics of the twenty-five T1DM, and 12 control subjects are displayed in Table 1.

**Table 1:** Characteristics of T1D and control subjects included in the study.

*Variables*	*T1D*	*Controls*	*p*
Number	25	12	---
Age (years)	38±13	37±9	0.74
Male (%)	76	75	0.41
Total cholesterol (mg/dL)	166±22	148±16	0.15
HDL-cholesterol (mg/dL)	55±12	59±11	0.52
Triglycerides (mg/dL)	75±34	69±10	0.84
Fasting plasma glucose (mg/dL)	148±64	72±7	<0.01
HbA1c (%)	7.6±0.7	---	---
SBP (mmHg)	125±13	111±7	<0.01
DBP (mmHg)	79±9	74±8	0.13
HR (bpm)	72±10	79±8	0.06
Body weight (kg)	73±11	65±9	0.19
Height (meter)	1.71±0.08	1.67±0.05	0.08
BMI (kg/m^2^)	24.6±3.1	23.1±2.3	0.11
Disease duration (year)	17±9	---	---
Total daily insulin (unit)	44±17	---	---
Insulin unit/kg body weight	0.6±0.2	---	---
MDI/Pump user (nr)	17/8	---	---

HbA1c: glycated hemoglobin; SBP: systolic blood pressure; DBP: diastolic blood pressure; HR: heart rate;

BMI: body mass index; MDI: Multiple Daily insulin Injection. Values are expressed as mean±SD and percentage.

Blood glucose and SBP were significantly higher in T1DM. Range of disease duration and HbA1c in T1DM was 3–33 years, and 6.3–8.8% (45–73 mmol/mol), respectively. Seventeen (68%) T1DM subjects were on multiple daily insulin injections, and 8 (32%) were on pumps. Nine (36%) T1DM subjects had non-proliferative diabetic retinopathy (2 moderate and 7 mild). No subjects had positive test for albuminuria or history of diabetic neuropathy.

Deep forearm flexor muscles parameters detected by CEU are displayed in Table 2.

**Table 2:** Vascular variables of forearm deep flexor muscles detected by CEU.

*Variables*	*T1D*	*Controls*	*p*
Number	25	12	---
Baseline-VI small vessels (a.u./mm^2^)	0.22±0.16	0.44±0.35	<0.05
Peak-VI small vessels (a.u./mm^2^)	0.33±0.24ǂ	0.72±0.52ǂ	<0.03
Time to peak-VI small vessels (s)	5.6±2.2	4.0±1.7	<0.02
Baseline-VI large vessels (a.u./mm^2^)	63.9±30.9	54.8±17.6	0.59
Peak-VI large vessels (a.u./mm^2^)	89.1±28.7ǂ	81.2±25.6ǂ	0.53
Time to peak-VI large vessels (s)	5.5±3.7	3.9±1.7	0.16

Values are expressed as mean±SD; VI: Video Intensity;

^ǂ^p<0.001 vs baseline-VI (matched pair test).

T1DM subjects had significantly lower VI at baseline and during reactive hyperemia compared with control subjects. However, VI significantly increased after ischemia in both groups. VI measured in larger vessels was not significantly different between T1DM and controls, but again significantly increased after ischemia in both groups. Interestingly, in T1DM subjects the time needed to reach peak-VI in small vessels was significantly longer than in control subjects. No difference was detected in time to peak-VI in larger vessels.

Table 3 shows brachial artery parameters and FMD for both groups.

**Table 3:** Brachial artery diameter, blood flow velocity and FMD of T1D and control subjects.

*Variables*	*T1D*	*Controls*	*p*
Number	25	12	---
Baseline brachial artery diameter (mm)	3.8±0.6	3.6±0.7	0.41
SPV (cm/s)	123±29	127±18	0.65
EDV (cm/s)	16±12	11±8	0.27
TAPV (cm/s)	35±14	34±17	0.89
Brachial artery blood flow (ml/min)	133±62	121±91	0.58
FMD 50s (% dilation)	5.4±6.4	10.7±4.5*	<0.01
Peak FMD (% dilation)	6.5±5.2	14.3±6.3*	<0.01
EarlyDilators (%)	48	83	0.90
Late Dilators (%)	32	17	0.90
No Dilators (%)	20	0	0.90

SPV: Systolic Peak Velocity; EDV: End Diastolic Velocity; TAPV: Time Average Peak Velocity; FMD: Flow Mediated Dilation; Values are expressed as mean±SD and percentage.

*p<0.05 vs T1D

Baseline diameter and blood flow velocities were comparable. FMD at 50s after ischemia and peak FMD were significantly lower in T1DM. The prevalence of Early dilators was higher in control subjects compared to T1DM subjects, while the prevalence of Late and No dilators was lower (none of the controls had No dilation vs 5 in T1DM group). However, the differences were not statistically significant.

T1DM with retinopathy had a significantly lower baseline-VI of small and large vessels, compared to T1DM without retinopathy. Peak-VI and the time to peak-VI of small and large muscle vessels were comparable between two groups (Table 4). The FMD calculated at 50s and peak FMD were lower in subjects with retinopathy compared with those without retinopathy, but the difference was not statistically significant. When control subjects (nr. 12) were compared with T1DM subjects without retinopathy (nr. 16) still a statistically significant difference between baseline-VI of small vessels (controls 0.44±0.35 vs T1DM without retinopathy 0.27±0.18 a.u./mm^2^; p = 0.02), and time to peak-VI of small vessels (controls 4.0±1.7 vs T1DM without retinopathy 5.6±2.3; p = 0.05) was observed.

**Table 4:** Vascular variables of forearm deep flexor muscles detected by CEU, and FMD of T1D subjects divided according to the presence or absence of retinopathy.

*Variables*	*T1D with retinopathy*	*T1D without retinopathy*	*p*
Number	9	16	---
Baseline-VI of small vessels (a.u./mm^2^)	0.13±0.10	0.27±0.18	0.03
Peak-VI of small vessels (a.u./mm^2^)	0.23±0.15	0.38±0.27	0.15
Time to peak-VI small vessels (s)	5.4±2.2	5.6±2.3	0.83
Baseline-VI of large vessels (a.u./mm^2^)	45±22	74±30	0.03
Peak-VI large vessels (a.u./mm^2^)	79±22	94±31	0.22
Time to peak-VI large vessels (s)	5.0±2.6	5.8±4.2	0.62
FMD 50s (% dilation)	3.3±4.5	6.4±7.1	0.28
Peak FMD (% dilation)	5.1±3.7	7.3±6.3	0.38

Values are expressed as mean±SD and percentage; VI: video intensity.

The simple correlation demonstrated a significant inverse relationship between baseline-VI of small vessels and age (r^2^–0.19; p<0.01), baseline-VI and baseline brachial artery diameter (r^2^–0.17; p<0.02), and baseline-VI and body weight (r^2^–0.24; p<0.003). Furthermore, a significant direct relationship was found between baseline-VI and peak FMD (r^2^ 0.17; p<0.02). Similar results were detected between peak-VI of small vessels and baseline brachial artery diameter (r^2^–0.14; p<0.03), peak-VI and body weight (r^2^–0.18; p<0.01), and between peak-VI and peak FMD (r^2^ 0.27; p<0.002). No significant relationship was found between baseline-VI of small vessels and baseline-VI of large vessels, and between peak-VI of small vessels and peak-VI of large vessels. As expected a key relationship was found between baseline-VI and peak-VI of small vessels (r^2^ 0.53; p<0.0001).

Finally, a multiple linear regression analysis was performed in order to evaluate variables significantly and independently correlated with VI measured at baseline and after ischemia. An inverse significant correlation between baseline brachial artery diameter and VI of deep forearm flexor muscle of small vessels at baseline, and a direct significant correlation between baseline brachial artery diameter and Delta VI of deep forearm flexor muscle small vessels were found.

**Fig 2:**
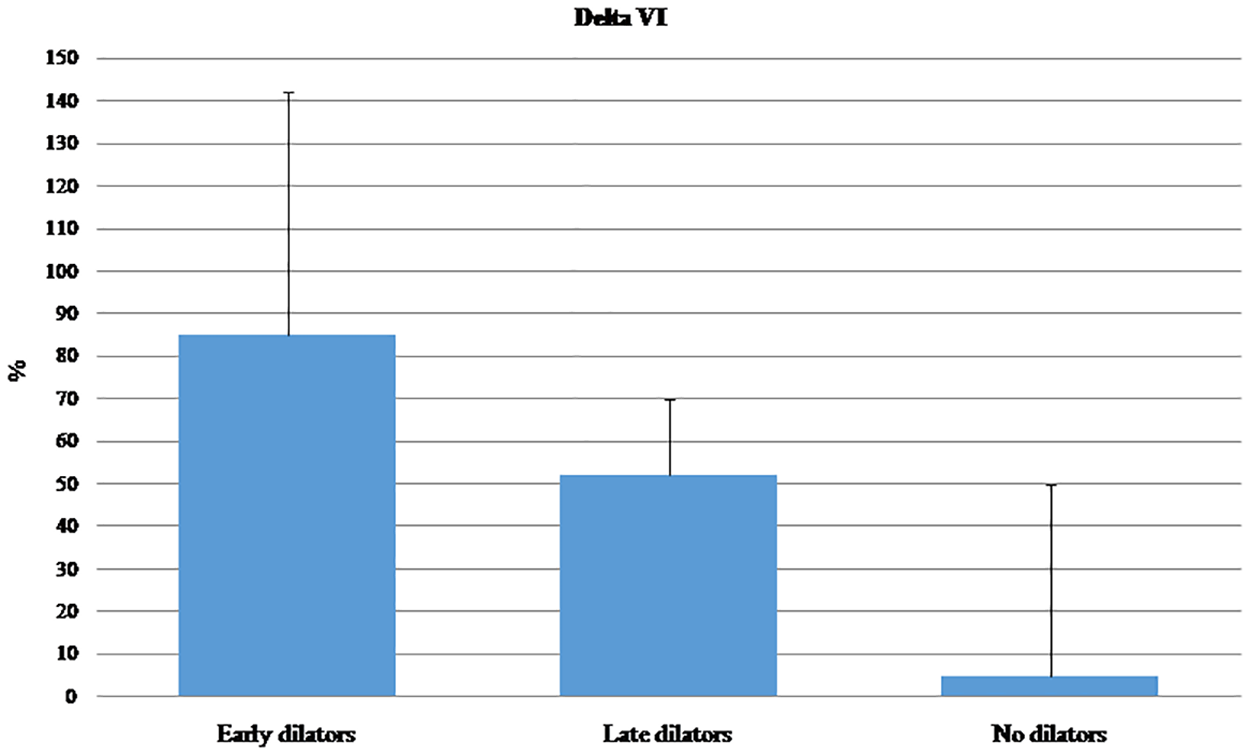
Delta VI of forearm deep flexor muscles small vessels in all subjects divided according to the time of maximal dilation.

Fig 2 shows the Delta VI measured at the small vessels of the muscles in all subjects recruited for the study and divided according to the time of maximal FMD. The Delta VI significantly decreased from Early to No dilation. The time to peak in VI of small vessels was 4.3±1.9 in Early dilators, 5.1±2.2 in Late dilators and 7.0±2.0 in No dilators. The p for trend was 0.07.

## Discussion

The present findings demonstrate that T1DM patients have defective peripheral skeletal muscle perfusion both at rest and after ischemia compared with control subjects. Low muscle perfusion associates with low FMD of the brachial artery, and with delayed or absent FMD. Furthermore, T1DM patients with retinopathy have the least muscle perfusion and a blunted response to hyperemia compared to T1DM without retinopathy.

Limb muscle tissue is particularly suitable for the study of the microcirculation for two main reasons: it is easily accessible to ultrasound and it is possible, through physical exercise or ischemia, to perturb its blood supply and observe responses. Under resting conditions not all of the muscle capillaries are patent. During exercise, or following ischemia, underperfused capillaries are recruited ensuring increased muscle perfusion. The degree of capillary recruitment is controlled by the dilation of terminal arterioles, that in turn dilate in proportion to the type/intensity of stimulation[24]. The number of patent capillaries influences tissue perfusion more than blood flow velocity. Indeed, blood velocity in capillaries is very slow and does not change significantly during exercise or in the post-prandial state[3,25–26].

CEU allows assessment of muscle perfusion in real time. The technique is highly sensitive with reasonable robustness[21]. CEU is minimally invasive, using microbubbles which resonate while stimulated by ultrasound with a low mechanical index. The quantification of backscattered ultrasound, expressed as Video Intensity, gives an estimate of capillary density.

Results displayed in Table 2 suggest that the deep forearm flexor muscle patent capillary density of T1DM patients in the present study may be only one-half that of control subjects, both at rest and after transient local ischemia. Since the patients were relatively young, fairly controlled and without additional cardiovascular risk factors, it can be assumed that chronic hyperglycemia per se might be responsible for reduced functional capillary density. Interestingly, both baseline small vessels perfusion and the time needed to increase perfusion after ischemia were still significantly lower and higher respectively in subjects with T1DM without retinopathy compared with control subjects. In other words, even subjects with uncomplicated diabetes might have a defective capillary perfusion.

Decreased muscle capillary density, assessed by morphometric analysis of biopsy samples, has been previously reported in subjects with type 2 diabetes. In that study the authors analyzed vastus lateralis muscle biopsies from young (mean age 24 years) and older subjects with diabetes (mean age 70 years) [27]. Others have reported that a progressive decline of muscle biopsy capillary density characterizes different levels of insulin resistance in older obese subjects[28]. Defective muscle perfusion in older subjects with diabetes or pre-diabetes is likely the effect of the combination of reduced muscle mass and anabolic insulin resistant state leading to impairment of microvascular function[29]. We have found, with a minimally-invasive technique, similar results but in young T1DM patients. While the above-mentioned anatomic characterizations are consistent with our results, we emphasize that CEU provides a functional measure of perfusion in vivo and also allows examination of physiologic responses to perfusion. These variables are not explorable in biopsy samples.

The non-invasive nature of our study does not allow us to understand whether the reduced VI both at rest and after ischemia is a functional and possibly transient impairment or a stable structural change, but the severe impairment detected in T1DM with retinopathy suggests a progressive structural change. Though fasting plasma glucose and HbA1c did not explain the reduced VI, the lack of association does not exclude that hyperglycemia may be the major determinant of vascular impairment. Glycemic variability as well as fluctuation in HbA1c might be responsible for defective microcirculation.

Amarteifio et al., using CEU, evaluated change in the microcirculation of the triceps surae muscle after 1 min of ischemia induced by a cuff placed around the thigh in subjects with severe claudication and moderate arterial stenosis in the lower limbs. They found depressed capillary recruitment during reactive hyperemia as well as delayed refilling after occlusion compared with healthy subjects[30]. The same authors have also investigated micro-perfusion of the triceps surae in 60-year old subjects with type 2 diabetes without peripheral arterial disease, finding that those with longer disease duration had impairment in VI replenishment curve following ischemia[15]. Our findings are in line with these results, but have been obtained in healthy young T1DM subjects, without additional cardiovascular risk factors.

A further relevant finding of the present study, in our opinion, is that the reduced response observed in small vessels correlates with a reduced response in the upstream artery. Indeed, the results in Table 5 describe the independent association between peak-VI of small vessels and peak FMD. As expected, and widely reported in literature, T1DM patients had impaired FMD compared with healthy subjects. We have further observed that the capillary density in T1DM significantly decreased in patients with Late and Absent FMD (Fig 2). Again, the nature of the study does not allow us to understand whether the alteration of the small vessels causes the impairment of large arteries or *vice versa* or whether both are reacting similarly to the diabetic state. Further studies are needed to investigate this aspect, possibly using the continuous measurement of FMD. In line with this observation, it must be also pointed out that the time to reach the maximum VI was markedly longer in T1DM, with particular regard to the microvasculature.

**Table 5:** Multiple regression analyses between baseline-VI and peak-VI of deep forearm flexor muscle small vessels in T1DM and control subjects.

*Baseline-VI of small vessels*	B unstandardized	B unstandardized SE	β	p
Age	-0.01	0.0001	-0.53	<0.01
Peak FMD	0.02	0.001	0.44	<0.02
*Peak-VI small vessels*	B unstandardized	B unstandardized SE	β	
Peak FMD	0.03	0.009	0.52	0.01

Independent variables included in the model were: age, baseline brachial artery diameter, peak FMD.

We have previously reported that delayed FMD of the brachial artery is associated with increased cardiovascular risk, increased prevalence of carotid atherosclerosis, increased carotid artery IMT, and acute cardiovascular events[17–20]. In the present study we have observed that T1DM patients who have Late dilation of the brachial artery also have a major impairment of capillary reactivity to ischemia.

In conclusion, the key messages of this study can be summarized in the following points: the CEU technique, currently principally used clinically to study liver and kidney lesions, may be a promising tool to evaluate muscle perfusion; young T1DM patients, even if apparently healthy and with acceptable HbA1c, likely harbor latent vascular dysfunction. Finally, the function as well as the dysfunction of microcirculation appears to correlate with (dys)function in larger vessels. The temporal continuum between the two beds should be further and carefully investigated.
